# Multi-omics dissection of tumor microenvironment-mediated drug resistance: mechanisms and therapeutic reprogramming

**DOI:** 10.3389/fphar.2025.1634413

**Published:** 2025-07-07

**Authors:** Fanghua Chen, Yuandong Fu, Gaigai Bai, Junjun Qiu, Keqin Hua

**Affiliations:** ^1^ Obstetrics and Gynecology Hospital of Fudan University, Shanghai Key Lab of Reproduction and Development, Shanghai Key Lab of Female Reproductive Endocrine Related Diseases, Shanghai, China; ^2^ Shanghai Key Laboratory of Female Reproductive Endocrine Related Diseases, Obstetrics and Gynecology Hospital of Fudan University, Shanghai, China

**Keywords:** tumor microenvironment, drug resistance, multi-omics, therapeutic strategies, drug delivery, immune evasion

## Abstract

Tumor drug resistance represents a major challenge in contemporary cancer therapeutics, significantly compromising the clinical efficacy of chemotherapy, targeted therapy, and immunotherapy. While existing research has elucidated the critical role of tumor cell-intrinsic mechanisms in drug resistance—including genomic instability, persistent activation of signaling pathways and aberrant epigenetic modifications—emerging evidence highlights the crucial involvement of dynamic remodeling within the tumor microenvironment (TME) in driving therapeutic resistance. The TME fosters drug resistance through dynamic remodeling, creating hypoxic conditions, immunosuppressive networks, and metabolic stress, which collectively impair treatment response and promote therapeutic escape. Advances in multi-omics technologies now enable a comprehensive, multi-dimensional analysis of these interactions, integrating genomic, epigenomic, transcriptomic, proteomic, and metabolomic data to uncover critical molecular networks and vulnerabilities. In this review, we explore the key mechanisms by which the TME influences drug resistance, discuss how multi-omics approaches enhance our understanding of these processes and evaluate emerging therapeutic strategies aimed at reprogramming the TME to overcome resistance.

## 1 Introduction

The tumor microenvironment (TME) dynamically orchestrates drug resistance through integrated cellular and molecular networks (Vennin et al., 2018; Albini and Sporn, 2007). Beyond cancer cell-intrinsic mechanisms, stromal components—including cancer-associated fibroblasts (CAFs), tumor-associated macrophages (TAMs), and other immunosuppressive cells—co-opt physiological processes such as metabolic symbiosis, extracellular matrix (ECM) remodeling and immune evasion to sustain tumor survival under therapy (Jain, 2013; Casey et al., 2015). This adaptive crosstalk results in spatially heterogeneous resistance niches, posing a fundamental challenge to current treatments.

While traditional approaches have identified individual resistance pathways, they fail to capture the TME’s systems-level complexity (Junttila and de Sauvage, 2013). The advent of multi-omics technologies now offers an unprecedented opportunity to dissect these complexities (He et al., 2023; Sun et al., 2023; Yan et al., 2025). By integrating genomic, transcriptomic, proteomic, and metabolomic data—complemented by spatial and single-cell resolution—researchers can map the multi-dimensional interactions between tumor cells and their microenvironment, revealing how these relationships drive resistance. This review synthesized current knowledge on TME-mediated drug resistance through a multi-omics lens, emphasizing key interactive networks (e.g., immune-stromal-metabolic axes) that sustain resistance, influencing factors (e.g., genetic change and epigenetic remodeling) uncovered via multi-omics, and emerging interventions targeting TME-omics vulnerabilities, including stromal reprogramming, immune-metabolic modulation, and combinatorial strategies.

## 2 Mechanisms of drug resistance mediated by TME

The TME orchestrates drug resistance through an intricate interplay of immunosuppression (Cappellesso et al., 2022; Pitt et al., 2016), physical drug delivery barriers (Yang et al., 2021; Stylianopoulos et al., 2018), metabolic reprogramming (Yang et al., 2020; Li et al., 2022; Zhang et al., 2025a) and aberrant intercellular communication (Lu et al., 2023; Rebelo et al., 2023; Liu et al., 2024). These mechanisms do not operate in isolation but rather form a coordinated defense network that enables tumors to evade therapeutic pressure. Understanding these processes is critical for developing strategies to overcome treatment resistance.

### 2.1 Cellular components of TME and drug resistance

#### 2.1.1 CAFs

At the cellular level, CAFs promote therapy resistance through ECM remodeling, generating dense fibrotic barriers that impede drug penetration. In pancreatic ductal adenocarcinoma (PDAC), for instance, TGFβ-high PDACs drive fibrosis, resulting in elevated collagen fiber density and tissue stiffness, which further restricts chemotherapeutic delivery (Hosein et al., 2020). Beyond physical obstruction, CAFs serve as a major source of cytokines, chemokines, and growth factors within the TME. By recruiting Tregs via CXCL12 and activating stromal stiffening pathways such as VAV2, CAFs reinforce immunosuppression—a mechanism linked to trastuzumab resistance in HER2+ breast cancer (Yang et al., 2016; Kieffer et al., 2020). CAFs could also secrete extracellular vesicles (EVs) in autocrine and paracrine signaling, enhancing cancer cell aggressiveness and therapeutic resistance. For instance, CAF-derived exosomal miR-423-5p promotes taxane resistance in prostate cancer by targeting GREM2 and amplifying TGF-β signaling, leading to increased proliferation and reduced apoptosis upon taxane exposure (Shan et al., 2020). Similarly, in colorectal cancer (CRC), CAFs-derived exosomes confer radioresistance by suppressing DNA damage and inhibiting apoptosis in CRC cells (Chen et al., 2021). Additionally, metabolic reprogramming of CAFs fosters clinical drug tolerance. For example, EGFR- or MET-expressing cancer cells enhance glycolytic activity and lactate production, stimulating CAFs to secrete HGF via NF-κB, thereby activating MET signaling and driving TKI resistance (Apicella et al., 2018).

#### 2.1.2 TAMs

Ideally, TAMs within the TME could theoretically eliminate tumor cells through phagocytosis and activate T-cell-mediated antitumor immunity, but their inherent plasticity and the complex TME often limit this therapeutic potential (Wang et al., 2025). For example, in advanced lymphoma, CD47-targeted therapy fails due to compromised macrophage phagocytic function within the TME (Cao et al., 2022).

M2-polarized TAMs exacerbate therapeutic resistance by secreting IL-10 and TGF-β, expressing PD-L1, and sequestering drugs, which collectively suppress cytotoxic T-cell activity and immunotherapy efficacy (Kanlikilicer et al., 2020; Shi et al., 2022; Croci et al., 2014). These macrophages also promote tumor vascularization through angiogenesis induction, basement membrane degradation, and secretion of pro-angiogenic factors such as VEGF and MMPs (MMP7, MMP9, MMP12) (Giraudo et al., 2004; Chryplewicz et al., 2022). VEGF reinforces M2-like polarization while collaborating with TAMs to disrupt vascular function, reducing drug perfusion and increasing treatment tolerance. In glioblastoma, bevacizumab-induced VEGF depletion unexpectedly elevates macrophage migration inhibitory factor (MIF) at the tumor periphery, expanding TAM infiltration (Castro et al., 2017). Lung adenocarcinoma TAMs similarly suppress p53 and PTEN while overexpressing VEGF-C/VEGFR3, inhibiting apoptosis and conferring doxorubicin resistance (Li et al., 2017). Macrophage-derived EVs mediate intercellular communication by transporting proteins, metabolites, and nucleic acids across the TME. The multidrug resistance protein P-glycoprotein (P-gp), encoded by MDR1, actively exports chemotherapeutics; in ovarian cancer, exosomal miR-1246 amplifies P-gp function through the Cav1/P-gP/PRPS2 axis, reducing paclitaxel uptake and accelerating chemoresistance (Kanlikilicer et al., 2020). Additionally, TAMs elevate glucose metabolism to stimulate OGT-dependent O-GlcNAcylation of Cathepsin B, enhancing its secretion into the TME and facilitating therapeutic evasion (Shi et al., 2022).

#### 2.1.3 Endothelial cells and immune cells

Tumor endothelial cells form abnormal, leaky blood vessels with heterogeneous permeability but inadequate perfusion, creating hypoxic niches that enhance cancer cell survival and therapy resistance. Hypoxia triggered by anti-VEGF therapy increases galectin-1 expression in tumor cells, where it binds glycosylated VEGFR2 on endothelial cells to sustain angiogenesis through VEGFA-mimetic signaling (Croci et al., 2014). Endothelial cells also develop acquired resistance to antiangiogenic tyrosine kinase inhibitors (Wu, S. et al., 2020) and chemotherapeutic agents (Huang et al., 2013a), employing mechanisms similar to tumor cells, such as P-gp upregulation (Akiyama et al., 2012). Functional reprogramming, phenotypic switching, and altered secretory profiles further enable endothelial evasion of antiangiogenic therapy.

Tregs accumulate in the TME through chemokine-mediated recruitment (e.g., CCL17/CCL22-CCR4, CCL28–CCR10, CCL5–CCR5) (Faget et al., 2011; Facciabene et al., 2011; Halvorsen et al., 2016) and peripheral conversion via TGF-β and IL-10 (Park et al., 2022). They suppress cytotoxic T cells by downregulating effector molecules such as granzyme B and IFN-γ (Yu et al., 2021; Huang et al., 2020), further compromising therapeutic efficacy. MDSCs suppress various immune cells, primarily targeting T cells, through the production of ARG1, iNOS, TGF-β, IL-10 and COX2, and also promote immunosuppression by inhibiting CD8^+^ T cell activity and inducing the differentiation of Tregs (Gabrilovich, 2017; Huang et al., 2006). In cisplatin-resistant bladder cancer, MDSCs are recruited via chemokine upregulation in response to cisplatin treatment and suppress CD8^+^ T cell responses through enhanced ARG1 and iNOS expression. Their accumulation not only promotes resistance to cisplatin but also impairs the efficacy of PD-L1 blockade, highlighting their role in mediating resistance to both chemotherapy and immune checkpoint inhibitors (ICIs) (Takeyama et al., 2020). These cellular interactions create a self-reinforcing cycle of immune suppression that undermines both conventional therapies and immunotherapies.

#### 2.1.4 Cancer stem cells (CSCs)

CSCs demonstrate dynamic plasticity by alternating between proliferative and quiescent states, with dormancy facilitating long-term survival through metabolic suppression while retaining cell cycle re-entry capacity under specific conditions (Vira et al., 2012). The biological adaptability makes quiescent CSCs especially refractory to cycle-dependent chemotherapeutics such as taxanes. Furthermore, their characteristic slow-cycling behavior, frequently involving extended G1 or S phase arrest, additionally confers resistance to diverse agents including cisplatin, taxol, and doxorubicin (Gao et al., 2010). A notable example is the Zinc Finger E-Box-Binding Homeobox 2 (ZEB2) in CRC, where its overexpression increases the proportion of CSCs in G0/G1 phase, directly contributing to platinum resistance (Francescangeli et al., 2020). Paradoxically, radiation therapy enriches CSC populations due to their inherent radioresistance relative to differentiated tumor cells. Radiation-resistant non-small cell lung cancer (NSCLC) cell lines exhibit marked upregulation of CSC markers like SOX2, CD133, and ALDH, with SOX2 critically enhancing DNA repair mechanisms and radioresistance (Qi et al., 2017). Glioblastoma studies similarly demonstrate that OXM1 induces SOX2 expression, amplifying radioresistance in this aggressive malignancy.

CSCs reside in specialized protective niches within the TME, particularly in perivascular and hypoxic zones, where stromal components, including CAFs and TAMs, sustain their survival and therapy resistance through diverse pathways. CSCs also maintain intricate bidirectional communication with immune cell populations. M2-polarized TAMs preserve CSC populations via chemokine secretion (e.g., IL-6, CCL2), activation of stemness pathways such as Wnt/β-catenin, and evasion of phagocytosis through CD47-SIRPα interactions (Liu et al., 2017; Ma et al., 2009). MDSCs reinforce immunosuppression by inhibiting T cell function while simultaneously enhancing CSC properties through STAT3 and Notch pathway activation (Ouzounova et al., 2017). CSCs systematically evade T cell recognition by downregulating antigen presentation components, losing MHC-I expression, inducing T cell tolerance, and secreting immunosuppressive cytokines like TGF-β and IL-10 (Rothstein and Sayegh, 2003). Although natural killer (NK) cells can target CSCs through cytotoxic mechanisms and differentiation induction, CSCs develop resistance strategies including ligand shedding, Treg recruitment, and modulation of activating receptor expression. While B cells and tertiary lymphoid structures (TLS) within the TME influence overall tumor prognosis and immune responses (Petitprez et al., 2020; Rodriguez et al., 2020), their specific interplay with CSCs requires further elucidation.

### 2.2 Biochemical and metabolic barriers in TME

#### 2.2.1 Hypoxia and acidosis

Hypoxia and acidosis represent two fundamental and interconnected features of the TME that significantly influence therapeutic outcomes. The landmark discovery of hypoxia-induced angiogenesis and subsequent elucidation of HIF-mediated transcriptional reprogramming revealed not only fundamental drivers of tumor progression but also major obstacles to successful cancer therapy. Notably, these hypoxia-driven adaptations promote chemoresistance through multiple mechanisms, including the upregulation of ATP-binding cassette (ABC) transporters such as ABCB1/P-gp, ABCC1, and ABCG2, which actively efflux diverse chemotherapeutic agents such as vinca alkaloids, anthracyclines, and platinum compounds (Dong et al., 2020; Li et al., 2016; He et al., 2016). Hypoxia simultaneously activates pro-survival pathways (e.g., Ras-MAPK, PI3K-Akt-mTOR) and stemness programs (e.g., Wnt, Notch), while suppressing apoptotic machinery through stabilization of anti-apoptotic proteins (e.g., IAP3, Bcl-2) and TP53 destabilization (Chen et al., 2023a; Kaloni et al., 2023; Cao et al., 2020). Tumor acidosis, another defining TME characteristic, mediates significant immunosuppression. Kreutz et al. established that proton and lactate exposure progressively compromises immune cell function, suppressing T cell secretion of IL-2, IFN-γ, granzyme B, and perforin while reducing monocyte-derived TNF (Sennino and McDonald, 2012). These observations demonstrate how the acidic TME fosters an immune-privileged environment conducive to tumor immune evasion.

#### 2.2.2 Nutrient deprivation and metabolic reprogramming

Metabolic reprogramming constitutes a fundamental cancer hallmark, allowing malignant cells to thrive in nutrient-scarce environments while developing therapeutic resistance through dysregulated glucose, lipid, and amino acid metabolism. The Warburg effect, marked by preferential aerobic glycolysis, depends on key regulatory enzymes such as hexokinase-2 (HK2), phosphofructokinase-1 (PFK1), and pyruvate kinase (PK). HK2 overexpression is associated with aggressive breast cancer phenotypes, and its pharmacological inhibition using 2-deoxy-D-glucose (2-DG) enhances the efficacy of conventional chemotherapeutics like doxorubicin and paclitaxel (Patra et al., 2013), with clinical studies confirming 2-DG’s chemosensitizing potential (Raez et al., 2013). Glutaminolysis also functions as a crucial anaplerotic pathway, sustaining both glycolytic and oxidative phosphorylation fluxes in cancer cells while maintaining redox balance and driving resistance to targeted therapies, as observed in HER2-positive cancers with reactivated mTOR signaling following lapatinib treatment (Deblois et al., 2016; Yang et al., 2017). Cancer cells display significant lipid metabolism alterations, including increased fatty acid oxidation regulated by carnitine palmitoyltransferase 1B (CPT1B) and activation of the JAK/STAT3-CPT1B-FAO axis, which reinforces stemness and chemoresistance—a metabolic shift particularly pronounced in cisplatin-resistant ovarian cancer (Wang et al., 2018; Yoshida, 2015). Parallel metabolic adaptations, including pyrimidine metabolism and IDO-mediated tryptophan catabolism, provide tumors with metabolic flexibility while creating an immunosuppressive niche (Balkwill et al., 2012; Brandacher et al., 2006). Notably, dysregulated pyrimidine metabolism, a recognized cancer hallmark, has emerged as a key modulator of immunotherapy response. Preclinical evidence demonstrates that inhibition of pyrimidine synthesis reduces the frequency of CTLA4^+^ T cells within the TME, suggesting a metabolic checkpoint that shapes immune evasion (Siddiqui and Ceppi, 2020). Further supporting this link, pyrimidine metabolism-related genes correlate with immunotherapy outcomes, highlighting their potential as a therapeutic target (Luo et al., 2022). The complex crosstalk among these metabolic pathways highlights the intricacies of tumor metabolism while revealing new therapeutic avenues for treatment-resistant malignancies.

### 2.3 Adaptive evolution of TME under therapy

The TME exhibits substantial plasticity in response to conventional and targeted therapies, developing complex resistance mechanisms through dynamic cellular and molecular adaptations. Chemotherapy often induces EMT, which upregulates drug-resistance genes and recruits immunosuppressive cell populations, creating a therapy-resistant niche (Glaviano et al., 2025). Radiation therapy similarly alters the immune landscape by enriching MDSCs and Tregs, fostering an immunosuppressive environment (Jarosz-Biej et al., 2019). Although reactive oxygen species (ROS) generation mediates radiation-induced cytotoxicity, hypoxia-driven ROS elevation activates a compensatory antioxidant response that induces cytoprotective autophagy, ultimately conferring radioresistance (Eales et al., 2016).

Immunotherapy encounters distinct challenges from the TME’s adaptive responses, particularly through compensatory upregulation of alternative immune checkpoints. In anti-PD-1-treated lung cancer models, T cells show significant TIM-3 overexpression following PD-1 blockade (Koyama et al., 2016). Similarly, prostate tumors responding to CTLA-4 inhibition exhibit elevated PD-L1 and VISTA expression in immune subsets and malignant cells, potentially explaining the limited efficacy of ipilimumab in this context (Gao et al., 2017; Le et al., 2014). Metabolic crosstalk between tumor and stromal cells further drives microenvironmental adaptation. Enhanced glycolysis and mitophagy alter drug pharmacokinetics and sustain energy homeostasis, reinforcing therapeutic resistance (Okoye et al., 2023; Wu, H. et al., 2020). Tumor cells dynamically rewire their metabolic networks to support proliferation while shaping an immune-evasive TME, a reprogramming that not only promotes survival but also compromises immunotherapy through diverse resistance mechanisms (Wu et al., 2021a). Consequently, the evolving interplay between tumor cells and their microenvironment during treatment necessitates longitudinal monitoring to optimize therapeutic strategies.

### 2.4 Genetic and epigenetic determinants of resistance

High tumor mutation burden (TMB) enhances tumor immunogenicity by generating neoantigens that facilitate T-cell recognition and tumor clearance, correlating with improved responses to ICIs. However, the predictive power of TMB is context-dependent, influenced by factors such as the mutational landscape and the efficiency of neoantigen presentation. For instance, in melanoma, the variable clinical outcomes following PD-1/PD-L1 blockade can be attributed, in part, to the differential kinetics between genomic mutation acquisition and the final steps of MHC-mediated antigen presentation (Goodman et al., 2017). Beyond genetic alterations, epigenetic dysregulation—including aberrant DNA methylation, histone modifications, and chromatin remodeling—plays a pivotal role in shaping immune cell function and tumor immune escape (Yoo and Jones, 2006). Key epigenetic regulators such as EZH2 and DNMT1 suppress antitumor immunity by limiting CD8^+^ T-cell infiltration, amplifying Treg activity, and downregulating MHC-I expression, thereby fostering an immune-privileged niche (Khodayari et al., 2023; Kundu et al., 2024).

The complexity of these interactions underscores why conventional single-target approaches often fail against TME-mediated resistance. The interconnected nature of these mechanisms - where physical barriers influence drug distribution, metabolic changes alter the therapeutic landscape, and immune suppression protects surviving tumor cells - demands integrated therapeutic strategies. Recent advances in multi-omics approaches are beginning to unravel these complex networks, revealing novel vulnerabilities that could be targeted to overcome the TME’s formidable defenses. As our understanding of these resistance mechanisms deepens, so too does the potential for developing more effective combination therapies that simultaneously target multiple aspects of the resistant TME.

## 3 Multi-omics technologies deciphering TME-mediated drug resistance

**FIGURE 1:**
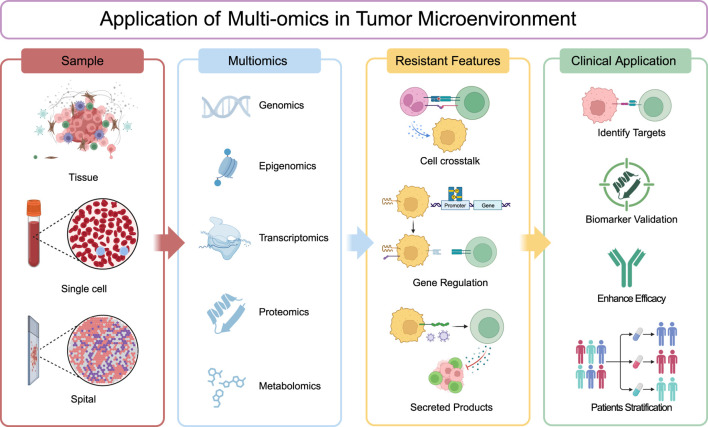
Application of multi-omics in tumor microenvironment. Tumor tissue samples are subjected to comprehensive analysis via single-cell and spatial profiling, as well as through various omics approaches including genomics, epigenomics, transcriptomics, proteomics, and metabolomics. These analyses can be conducted independently or integrated to provide a holistic view. The data obtained are then meticulously evaluated to uncover resistant features within the tumor microenvironment, such as cell-to-cell communication, gene regulatory mechanisms, and the secretion of bioactive molecules. The insights gleaned from these analyses are subsequently translated into clinical applications aimed at identifying therapeutic targets, validating biomarkers, enhancing treatment efficacy, and facilitating patient stratification for tailored therapeutic interventions.

Multi-omics technologies (genomics, epigenomics, transcriptomics, proteomics and metabolomics) now enable comprehensive mapping of these resistance networks across biological scales. As we explore specific omics applications in subsequent sections, this systems biology framework will highlight how multi-omics uncovers resistant mechanisms and latent therapeutic vulnerabilities (Figure 1), revealing the convergent mechanisms by which localized perturbations propagate into system-wide treatment failure.

### 3.1 Genomic alterations shape TME adaptation and reveal therapeutic vulnerabilities

Genomics has enabled the precise detection of single nucleotide variants, copy number variations and structural variations at single-cell resolution through whole-genome sequencing (WGS) and whole-exome sequencing (WES) technologies, revealing how genomic heterogeneity shapes TME evolution (Sherman and Salzberg, 2020; Gawad et al., 2016). Tumor-intrinsic alterations create dual therapeutic barriers by concurrently activating oncogenic pathways and sculpting an immunosuppressive niche. In NSCLC, epidermal growth factor receptor (EGFR) mutations identified by genomics not only sustain tumor cell survival but also correlate with an immune-cold TME phenotype characterized by low TMB and elevated PD-L1 expression (Madeddu et al., 2022), explaining the limited efficacy of PD-1 blockade in this subset. This genomic-immune axis was clinically validated in KEYNOTE-789 (NCT03515837), where pembrolizumab-chemotherapy failed to improve outcomes in EGFR-mutant NSCLC (Yang et al., 2024a). Similarly, WES identified KRAS/MEK1 compensatory alterations driving resistance to RAF/EGFR inhibition (Ahronian et al., 2015), prompting ongoing evaluation of triplet therapy (BRAF+EGFR+MEK) in NCT05217446. Beyond tumor cells, stromal genomic alterations also contribute substantially to therapeutic resistance. In PDAC, SMAD4-deficient CAFs activate a TGF-β/IL-11 signaling axis that drives stromal fibrosis and chemoresistance (Matsumura et al., 2022). These findings have spurred clinical exploration of stromal-targeting strategies, including the TGF-β receptor inhibitor galunisertib, which suppresses SMAD phosphorylation and disrupts canonical TGF-β signaling. A phase Ib study (NCT02734160) evaluating galunisertib plus the anti-PD-L1 antibody durvalumab in refractory metastatic PDAC suggests that TGF-β pathway inhibition may potentiate immunotherapy efficacy in this setting.

Collectively, these insights underscore the necessity of integrating tumor and stromal genomic profiling to fully decipher resistance mechanisms and optimize therapeutic strategies. Serial monitoring of clonal evolution—such as tracking trunk mutations (reflecting TMB) and resistance-associated variants (e.g., RAS/BRAF) in circulating tumor DNA (ctDNA)—further enables dynamic assessment of therapeutic vulnerability, as demonstrated in metastatic colorectal cancer (Vidal et al., 2023).

### 3.2 Epigenomic regulation of therapy resistance in the TME

Recent advances in epigenomic profiling, including chromatin immunoprecipitation sequencing (ChIP-seq) and transposase-accessible chromatin using sequencing (ATAC-seq), have elucidated how DNA methylation, histone modifications, and chromatin remodeling dynamically regulate tumor evolution and therapy resistance independent of genetic alterations (Weichenhan et al., 2022). Single-cell epigenomic analyses reveal direct links between epigenetic silencing and treatment failure - for instance, chemoresistant stem-like populations in relapsed pediatric AML exhibit reduced chromatin accessibility (Lambo et al., 2023). Emerging technologies like EPIC-seq further demonstrate the translational potential of epigenomics, enabling noninvasive cancer subtyping and prediction of PD-(L)1 inhibitor response through cfDNA-based promoter fragmentation entropy analysis (Esfahani et al., 2022). Epigenomics has found that hypomethylation promotes tumor cell resistance to treatment by affecting gene expression, signaling pathway activity, and epigenetic regulation. In gliomas, CAV1 demethylation confers resistance to oxidative phosphorylation inhibitors (Liu et al., 2023), while clinically, hypomethylating agents show promise for overcoming resistance in hematologic malignancies, as evidenced by the encouraging activity of oral decitabine/cedazuridine plus venetoclax in a recent phase 1/2 trial (NCT04655755). Super-enhancer remodeling represents another conserved resistance mechanism. Integrative analysis of H3K27ac ChIP-seq and RNA-seq data in microsatellite-stable CRC identified KLF3 as a critical regulator of chemoresistance via ABCB1/MDR1 activation (Li et al., 2021), suggesting potential for epigenetic-targeted combination strategies. These findings highlight epigenetic plasticity as a critical determinant of TME-driven resistance, highlighting the need for integrated approaches combining functional epigenomics, CRISPR-based screens, and clinical epigenome editing to develop next-generation therapeutic strategies.

### 3.3 Transcriptomic insights into the multilayered resistance mechanisms in the TME

Transcriptomic technologies have revolutionized our understanding of therapy resistance by decoding the dynamic interplay between tumor cells and their microenvironment across multiple resolution scales. Bulk RNA sequencing remains a cornerstone for clinical biomarker discovery, offering cost-effectiveness and compatibility with archival specimens. Computational deconvolution algorithms now enable precise reconstruction of TME composition from bulk data, identifying PD-1+ CD8^+^ T cells as predictive biomarkers of immunotherapy response—potentially refining clinical decision-making.

High-resolution single-cell sequencing (scRNA-seq) has further uncovered tumor-intrinsic and stromal mechanisms of resistance. In NRAS-mutant melanoma, scRNA-seq revealed rapid upregulation of the purinergic receptor P2RX7 within 72 h of MEK/CDK4/6 inhibition, promoting survival via calcium influx (Randic et al., 2023). This aligns with preclinical evidence that P2RX7 blockade enhances anti-PD-1 efficacy through IL-18-mediated NK and CD4^+^ T cell activation (Douguet et al., 2021). scRNA-seq has also exposed critical stromal contributions. In soft tissue sarcomas, a glycolytic CAF subset drives immunosuppression through GLUT1-mediated metabolic competition and CXCL16-dependent CD8^+^ T cell exclusion, spurring the development of GLUT1 inhibitors (Broz et al., 2024). In PDAC, LRRC15+CAFs form tumor-peripheral barriers absent in normal tissue, correlate with anti-PD-L1 resistance in 600+ patients, and represent a TGFβ-dependent therapeutic target (Dominguez et al., 2020).

Spatial transcriptomics has added an architectural dimension to resistance mechanisms. In triple-negative breast cancer (TNBC), three distinct immune microenvironments dictate therapeutic outcomes: (1) immunoreactive (intratumoral CD8+/IDO+), (2) immune-cold (B7-H4+/fibrotic stroma), and (3) immunomodulatory (stromal PD-L1+/cholesterol-rich) (Shiao et al., 2024). Immune-cold tumors exhibit pronounced checkpoint inhibitor resistance while macrophage-targeting therapies like bexmarilimab show context-dependent efficacy—activating “cold” TMEs but suppressing IFN-rich niches, as demonstrated in patient-derived explant cultures (Rannikko et al., 2025). A pan-cancer stromal atlas further delineated 39 functionally distinct subsets across 16 malignancies, revealing conserved resistance mechanisms: PGF+ endothelial tip cells mediate immune exclusion, while boundary CAFs drive LGALS9/TIM-3-dependent immune evasion (Du et al., 2024). Integrated single-cell and spatial transcriptomics in rectal cancer, CAF polarization dictates chemotherapy response, with tumor-suppressive CAFs organizing protective immune networks and FAP+ CAFs driving EMT (Qin et al., 2023). This multi-scale transcriptomic framework - from bulk biomarkers to single-cell dynamics and spatial architectures - provides an integrated roadmap for overcoming TME-mediated resistance through precision target identification, patient stratification, and rational combination therapies.

### 3.4 Metabolic reprogramming in the TME: a key driver of drug resistance

Recent advances in metabolomics have revolutionized our understanding of the TME, revealing how metabolic rewiring drives therapy resistance. Cutting-edge technologies—including liquid chromatography-tandem mass spectrometry (LC-MS/MS), matrix-assisted laser desorption/ionization mass spectrometry imaging (MALDI-MSI), and nuclear magnetic resonance (NMR) spectroscopy—now enable spatially resolved metabolic profiling, uncovering dynamic adaptations that sustain tumor survival under therapeutic pressure (Wishart, 2019). Metabolomic signatures have emerged as powerful predictors of drug response. Chemoresistance has been linked to specific metabolic alterations, including elevated levels of hypotaurine, uridine, dodecanoylcarnitine, choline, dimethylglycine, niacinamide, and L-palmitoylcarnitine (Tian et al., 2018). Similarly, metabolic profiling of patient-derived xenografts has identified resistance biomarkers for multiple anticancer agents (Kaoutari et al., 2021). In TNBC, glutamine accumulation sustains glutathione synthesis while NAD+ depletion impairs DNA repair, driving chemotherapy resistance (Carneiro et al., 2023). Then, it also led to the preclinical trial of glutaminase inhibitor CB-839 with platinum-based chemotherapy (Shen et al., 2020). In diffuse large B-cell lymphoma, MALDI-MSI identified resistant niches characterized by elevated AMP/ATP ratios and phosphatidylinositol accumulation (Raez et al., 2013), which guided the development of the PI3Kδ inhibitor, which normalized CD4/CD8 ratios and maximized the number of CD8^+^ T-stem cell memory, naive, and central memory T-cells with dose-dependent decreases in expression of the TIM-3 exhaustion marker (Funk et al., 2022). Similarly, in bladder cancer, LC-MS/MS revealed that HK2-mediated glycolytic flux drives cisplatin resistance, prompting the development of the HK2 inhibitor, which synergized with sorafenib to inhibit tumor growth (Gong et al., 2025; DeWaal et al., 2018).

Spatially resolved metabolomics has been instrumental in mapping tumor-stroma interactions and drug distribution. In glioblastoma, the oncometabolite D-2-hydroxyglutarate (D-2-HG), produced by IDH-mutant tumor cells, is taken up by CD8+ T cells, altering their metabolism and impairing cytotoxicity (Notarangelo et al., 2022). Multimodal imaging confirmed that D-2-HG-rich tumor regions exhibit cytotoxic T-cell exclusion, suggesting differential immunotherapy responses based on D-2-HG levels (Altea-Manzano et al., 2023). By elucidating the metabolic basis of resistance—metabolomics has become indispensable for developing precision therapies. Its integration into multi-omics frameworks promises to overcome TME-mediated treatment failure, offering new avenues for patient stratification and rational combination strategies.

### 3.5 Proteomic profiling of TME-driven resistance: networks and post-translational modifications

Proteomic technologies have emerged as indispensable tools for dissecting the molecular mechanisms underlying therapeutic resistance in cancer. By characterizing protein abundance, post-translational modifications (PTMs), and protein-protein interactions, proteomics provides a functional readout of cellular states that complements genomic and transcriptomic analyses. Recent advances in MS-based proteomics and spatial proteomics have revealed intricate networks of resistance mechanisms operating through tumor-intrinsic pathways and microenvironmental crosstalk. In PTEN-null prostate cancer, proteomic analyses have uncovered multifaceted resistance mechanisms involving MEK-dependent immunosuppressive TAMs and Wnt/β-catenin-driven metabolic adaptation. These findings directly informed the development of combination therapies targeting MEK/Wnt with PI3K inhibitors, achieving complete and durable responses in preclinical models (Chaudagar et al., 2023). Integrated lipidomics-proteomics approaches further identified a FABP4/SCD1-mediated redox maintenance circuit sustained by lipid droplet formation, offering both prognostic biomarkers and therapeutic targets to prevent recurrence (Luis et al., 2021).

While early studies primarily cataloged protein abundance changes—such as EphA2 enrichment in gemcitabine-resistant PDAC (Fan et al., 2018), or mitochondrial Complex I upregulation in trastuzumab-resistant HER2+ breast cancer (Tapia et al., 2024)—contemporary proteomics has shifted focus to PTMs as direct mediators of resistance. First, direct modulation of drug-target interactions occurs, such as hyper-N-glycosylation of PD-L1 in melanoma, which masks checkpoint inhibitor binding sites. This has motivated clinical trials combining PD-1 inhibitors with glycosylation modulators (Loison et al., 2024). Second, by rewiring the function of the protein, hypoxic conditions in ovarian cancer induce PIAS4-mediated SUMOylation of KDM5B, preventing its ubiquitin-dependent degradation and maintaining chemoresistance (Gu et al., 2023), which promotes the development of novel agents, such as the SUMOylation inhibitor TAK-981. In addition, USP5-mediated MDH2 deubiquitination drives ripretinib resistance in gastrointestinal stromal tumors (Wang et al., 2023), while the OTUD6A-CDC6 deubiquitination axis promotes bladder cancer chemoresistance (Cui et al., 2024). Third, the facilitation of intercellular crosstalk is another mechanism. Phosphoproteomic analyses have revealed resistance mechanisms, including EGFR/HMAG1 hyperphosphorylation and SRC-PRKCD cascade activation in EGFR inhibitor-resistant NSCLC. IL-6-induced STAT3 phosphorylation upregulates BCL-2 in breast cancer, conferring radioresistance and justifying JAK2 inhibitor trials (ruxolitinib, NCT04418154). Lactylation has emerged as a critical PTM in immune evasion. In CD8+ T cells, IL-11 activates JAK2/STAT3 to drive immune checkpoint expression via lactylation, promoting exhaustion (Zhou et al., 2024). KRAS-mutant tumors exhibit elevated histone lactylation (H3K18la) in tumor-specific CD8+ T cells, while p300-catalyzed APOC2 lactylation in NSCLC induces extracellular lipolysis and Treg accumulation, driving immune checkpoint blockade (ICB) resistance (Chen et al., 2024). Proteomic technologies have transformed our understanding of therapy resistance by revealing the dynamic post-translational landscape of cancer cells and their microenvironment. The integration of proteomic data with other omics layers and clinical outcomes promises to accelerate the development of effective combination therapies to overcome treatment resistance.

### 3.6 Integrative multi-omics approaches to decode TME-Mediated resistance

The multifaceted nature of tumor drug resistance necessitates a paradigm shift from single-omics to integrative multi-omics approaches. While genomics identifies driver mutations, transcriptomics reveals dynamic gene expression patterns, proteomics characterizes functional protein networks, and metabolomics uncovers metabolic adaptations, none of these layers alone can fully capture the complexity of treatment resistance. Integrative multi-omics strategies bridge this gap by systematically connecting molecular alterations across biological scales, offering unprecedented insights into the TME’s adaptive responses.

In esophageal cancer, combining genomic, transcriptomic, proteomic, and metabolomic data with machine learning uncovered three clinically relevant findings: DNA repair deficiencies predicting chemoradiotherapy response, glutaminolysis-driven metabolic rewiring underlying resistance, and immune-evasive phenotypes detectable only through integrated protein/RNA profiling (Yang et al., 2024b). Integrated scRNA-seq and single-cell ATAC-seq in tamoxifen-resistant breast cancer delineated tumor heterogeneity, revealing distinct cancer cell states, a core resistance gene signature, and BMP7’s role in oncogenic pathway modulation (Fang et al., 2024). In PDAC, combined scRNA-seq, metabolomics, and flow cytometry exposed chemotherapy-resistant TAMs that evade treatment via altered deoxycytidine metabolism, suggesting new therapeutic targets (Zhang et al., 2023a). Multi-omics profiling in HR+/HER2− metastatic breast cancer further identified APOBEC mutation patterns and homologous recombination deficiency-high clusters linked to CDK4/6 inhibitor resistance, refining patient stratification (Park et al., 2023).

Spatial multi-omics has been particularly transformative. In hepatocellular carcinoma (HCC), combined spatial transcriptomics and proteomics uncovered CXCL12^+^ tumor-associated endothelial cells that mediate immunosuppression by recruiting MDSCs and inhibiting CD8^+^ T cells - a resistance mechanism missed in bulk analyses (Lu et al., 2025). NSCLC studies merging scRNA-seq with spatial transcriptomics distinguished tertiary lymphoid structure states and tumor-stroma interactions predictive of chemoimmunotherapy response (Yan et al., 2025). Such spatial insights are vital for designing targeted combination therapies. Prostate cancer research further highlights multi-omics integration: single-cell analyses identified EpCAM-negative circulating tumor cells with unique androgen receptor (AR)-driven biology (Lambros et al., 2018), while spatial approaches revealed KLK3-mediated micrometastatic niches (Heidegger et al., 2022) and CXCL12-driven angiogenesis through combined scRNA-seq and functional assays. These discoveries are already informing clinical applications, including liquid biopsy platforms for AR-variant detection, KLK3-targeted adjuvant strategies, and trials evaluating CXCL12 inhibition. The clinical impact of multi-omics integration is becoming evident across multiple cancers. The gastric cancer study employed WES, whole-transcriptome sequencing and scRNA-seq to show that higher pre-treatment CD3^+^ T cell infiltration predicts poor response to neoadjuvant PD-1 inhibitors combined with chemotherapy (Ji et al., 2024). Bladder cancer studies identified BCAT2 as a key regulator of noninflamed TME, enabling novel ICB combinations (Cai et al., 2023). The cholangiocarcinoma study integrated analysis of 85 PANoptosis-related genes to uncover POSTN+ CAFs as key resistance orchestrators spatially linked to immunosuppressive TAMs and PD-L1/2, while developing the clinically actionable PANRS model (POSTN/SFN/MYOF/HOGA1/PECR) (Yu et al., 2025). TNBC analyses of 465 samples defined three metabolic subtypes with distinct therapeutic vulnerabilities, guiding subtype-specific strategies from lipid metabolism inhibitors to glycolytic blockade combined with anti-PD-1 immunotherapy (Gong et al., 2021). Glioblastoma studies leveraging multi-omics implicated PKCδ and DNA-PK as master kinases driving molecular subtypes, prompting an RNA-based classifier for clinical stratification and kinase inhibitor trials (Migliozzi et al., 2023). Comprehensive multi-omic profiling of hyperthermia-treated ovarian cancer cells uncovered rapid CDK1 hyperactivation, prompting the rational combination of WEE1 inhibitors with hyperthermic intraperitoneal chemotherapy to improve outcomes (Yang et al., 2024c). Similarly, a landmark study combining single-cell and spatial transcriptomics with bulk sequencing identified CXCL12^+^ CAFs as key architects of chemoresistance in ovarian cancer, leading to a clinically actionable 24-gene predictive signature (Wang et al., 2024a). These findings exemplify how integration provides insights unattainable through single-omics approaches.

## 4 Therapeutic strategies targeting the TME to overcome drug resistance

The intricate interplay between cellular and non-cellular components within the TME has necessitated the development of multifaceted therapeutic approaches to combat drug resistance. Recent advances in our understanding of TME biology, coupled with insights from multi-omics analyses, have enabled the design of precision strategies targeting key resistance mechanisms while preserving normal tissue function (Table 1).

**TABLE 1:** Therapeutic strategies targeting the TME to overcome drug resistance: Mechanisms, agents, and clinical progress.

Intervention strategy	Mechanism	Representative agents/Therapies	Clinical progress	Challenges/Limitations	References
CD25^+^ Treg-depleting antibody therapy	Depletes intratumoral Tregs to relieve immunosuppression	RG6292 (Vopikitug)	Phase I (NCT04158583)	Tumor heterogeneity; limited PD data	Belli et al. (2024)
CCR8^+^ Treg-depleting antibody therapy	Depletes CCR8^+^ Tregs to relieve immunosuppression	RO7502175	Phase I ongoing (NCT05581004)	Expression heterogeneity; ADA risk; safety unknown	Gampa et al. (2024)
CAF-targeted immunomodulation via FAP–4-1BB	Activates T cells through FAP-anchored 4-1BB costimulation	RO7122290	Phase I completed	Immune AEs; weak monotherapy response	Melero et al. (2023)
Inhibition of CAF-activating Hedgehog signaling	Blocks SMO to reduce CAF activation and improve drug delivery	Vismodegib (GDC-0449)	Approved for BCC; failed PDAC trial (NCT01130142)	Variable efficacy; potential immunosuppression	Sekulic et al. (2012)
Inhibition of CAF-induced CSC plasticity via Hedgehog signaling	SMOi blocks CAF activation and FGF5/collagen production, reducing CSCs and chemoresistance	Sonidegib + docetaxel	Phase I (EDALINE, NCT02027376) in TNBC	Response limited to tumors with high stromal Hh activity	Cazet et al. (2018)
TAM repolarization and depletion	CD40 agonist activates APCs; CSF1R inhibitor depletes M2 TAMs	APX005M + cabiralizumab ± nivolumab	Phase I (NCT03502330) in ICI-resistant tumors	Limited efficacy; cytokine spikes; timing concerns	Gomez-Roca et al. (2019)
CSF-1R inhibition	Depletes M2-like TAMs and reduces immunosuppression	Pexidartinib + paclitaxel	Phase Ib; RP2D established	Modest efficacy; liver toxicity	Wesolowski et al. (2019)
Macrophage reprogramming via PI3Kδ inhibition	PI3Kδ inhibitor reprograms TAMs, increasing M1 macrophage polarization and enhancing T cell activation	Eganelisib (IPI-145) + nivolumab	Phase I/II in mTNBC (NCT02646748)	Modest monotherapy efficacy; requires a combination for optimal effect	O’Connell et al. (2024)
CXCR4 inhibition to reduce MDSCs	Blocking CXCR4–CXCL12 axis to reduce MDSCs and boost T cells	BL-8040 + pembrolizumab ± chemo	Phase IIa (NCT02826486) in PDAC	Modest effect; small cohort; needs validation	Bockorny et al. (2020)
IL-1β and PD-1 dual blockade to reduce MDSCs	Decreases circulating Mo-MDSCs and modestly enhances CD8^+^ T cell activation	Canakinumab + spartalizumab + gem/nab-paclitaxel	Phase I (NCT04581343) in metastatic PDAC	Limited tumor MDSC response; small sample size	Oberstein et al. (2024)
VEGF blockade to reverse MDSC-mediated immunosuppression	Bevacizumab reduces intratumoral MDSCs and improves T-cell infiltration when combined with PD-L1 blockade	Atezolizumab + bevacizumab	Phase II (IMmotion150) in mRCC	Limited benefit in Myeloid^High tumors; needs biomarker stratification	McDermott et al. (2018)
CAF reprogramming to improve drug delivery	AM80 induces Meflin^+^ CAFs, softens stroma, enhances perfusion	AM80 (Tamibarotene) + chemo	Phase I/II (NCT05064618)	CAF plasticity; no direct anti-tumor effect	Mizutani et al. (2022)
MDR1 inhibition to overcome ADC resistance	Inhibits MDR1, restoring drug accumulation in cells	Cyclosporine A (CsA) + Brentuximab vedotin	Phase I (NCT03534561) in R/R Hodgkin lymphoma	Toxicity (myelosuppression); incomplete response in some patients	Chen et al. (2020)
Inhibition of Warburg effect in HCC	ASPP2 depletion enhances glycolysis via WNT/β-catenin/HK2 axis	2-Deoxyglucose (2-DG)	Preclinical (HCC models)	Requires clinical validation; challenges in clinical translation	Liang et al. (2023b)
Targeting GDF-15 to overcome immune checkpoint blockade resistance	GDF-15 inhibits T-cell infiltration and activation, preventing an effective immune response	Visugromab (anti-GDF-15) + nivolumab	Phase 1–2a (NCT04725474) in relapsed/refractory cancers	Limited patient cohort; response varies by tumor type	Melero et al. (2025)
Reprogramming the TME with epigenetic agents + PD-1 blockade	Inhibiting immunosuppressive IL21+ Th cells and reactivating tumor-specific CD8^+^ T cells	Chidamide + Decitabine + Camrelizumab (CDP)	Phase 2 (NCT04233294) in relapsed/refractory cHL	Patient heterogeneity; requires further validation in large cohorts	Nie et al. (2024)
Combination of CTLA-4 and PD-1 blockade	Reverses primary resistance to PD-1 blockade by enhancing T-cell infiltration and activity	Ipilimumab + Nivolumab	Phase 2 (NCT03033576) in metastatic melanoma	Increased toxicity (grade 3+ events); small sample size	VanderWalde et al. (2023)

### 4.1 Targeting cellular components of the TME

#### 4.1.1 CAFs

The immunosuppressive cellular network in the TME provides several potential therapeutic targets. Multi-omics studies have characterized CAF heterogeneity and their dynamic crosstalk with immune cells, informing two refined intervention approaches: direct CAF depletion and disruption of CAF-mediated immunosuppressive signaling. Early CAF depletion efforts focused on broad markers like FAP, α-SMA, and PDGFR (Qin et al., 2023; Grout et al., 2022). While FAP-directed CAR T cells and vaccines effectively eliminated FAP+ CAFs, increased CD8^+^ T-cell infiltration, and decreased fibrotic stroma (Kieffer et al., 2020; Wehrli et al., 2024; Pure and Blomberg, 2018; Wang et al., 2014), their clinical application was limited by on-target/off-tumor toxicity due to FAP expression in healthy tissues (Tran et al., 2013). Multi-omics data demonstrate that specific CAF subpopulations engage in reciprocal interactions with immune cells; for example, Cluster 0 FAP+ CAFs elevate PD-1 and CTLA-4 expression in Tregs, which subsequently drive the proliferation of immunosuppressive Cluster 3 CAFs associated with immunotherapy resistance (Kieffer et al., 2020). Spatial transcriptomics revealed that PDGFRβ+ CAF clusters recruit PMN-MDSCs through CXCL secretion, and disrupting these niches enhanced anti-PD-1 responses (Akiyama et al., 2023). Recent multi-omic profiling has uncovered CAF-specific surface markers including CD10, GPR77, POSTN, and LRR15, enabling more precise depletion strategies (Su et al., 2018; Purcell et al., 2018; Wang et al., 2024b; Krishnamurty et al., 2022). These findings underscore the need for more precise CAF-targeting strategies that account for functional heterogeneity and microenvironmental crosstalk, prompting recent efforts to develop subtype-specific interventions by multi-omic analysis.

An alternative strategy focuses on blocking CAF-derived immunosuppressive pathways, with multi-omics studies implicating TGF-β signaling, the IL-6/JAK/STAT3 axis, and chemokine networks. Clinical trials have demonstrated benefits with TGF-β inhibitors and bispecific anti-PD-L1/TGF-β antibodies (Kalluri, 2016; Faivre et al., 2019; Melisi et al., 2018; Ravi et al., 2018). IL-6R blockade enhanced CD8^+^ T-cell activation and synergized with checkpoint inhibitors (Heichler et al., 2020; Dijkgraaf et al., 2015), while CCR2 and CXCR4 inhibition improved therapeutic outcomes when combined with other agents (Xiang et al., 2020; Zhang et al., 2023b). Combined targeting of TGF-β signaling to prevent fibroblast activation and the CXCL12-CXCR4 axis to mitigate immune exclusion has proven particularly effective in restoring T-cell infiltration and potentiating checkpoint inhibitors (Rivas et al., 2022; Mariathasan et al., 2018; Feig et al., 2013). Emerging evidence also supports NOX4 inhibition as a viable approach, as it reverses CAF differentiation, promotes fibroblast normalization, and alleviates CD8^+^ T-cell exclusion to enhance anti-PD-1 responses (Ford et al., 2020).

#### 4.1.2 MDSCs

MDSCs represent a critical therapeutic target in cancer immunotherapy. Multi-omics profiling has identified four key intervention strategies: disrupting MDSC recruitment, depleting existing populations, inducing differentiation, and neutralizing their immunosuppressive mechanisms. Chemokine networks play a central role in MDSC trafficking, as demonstrated by single-cell RNA sequencing and immune profiling showing that PPARγ-mediated VEGF-A upregulation drives MDSC expansion through the PPARγ/VEGF-A axis (Xiong et al., 2023). Clinical evidence confirms bevacizumab reduces MDSC frequencies in both NSCLC and glioblastoma patients (Koinis et al., 2016; Peereboom et al., 2019). The CCL2-CCR2 axis further contributes to immune suppression, with CCR2 inhibitors like CCX872 showing promise in combination with PD-L1 blockade for HCC (Liang et al., 2023a; Xie et al., 2023). Metabolic vulnerabilities in MDSCs have emerged through multi-omics studies, including gemcitabine’s selective depletion of MDSCs while enhancing NK cell activity via NKG2D ligand upregulation. Cryo-thermal therapy similarly activates NK cells to eliminate MDSCs through the NKG2D-NKG2DL axis while IFN-γ drives their maturation, converting immunosuppressive MDSCs into immunostimulatory phenotypes (Peng et al., 2022). Multi-omics approaches have also identified hypoxic tumor-associated MDSCs that could be activated by the IL6/STAT3 pathway. Tyrosine kinase inhibitors such as sunitinib and cabozantinib have been shown to suppress MDSCs and sensitize tumors to CAR-NK cells (Zhang et al., 2017). Ongoing multi-omics investigations continue to reveal novel targets, including recent metabolomic findings that GLUT1-mediated glucose accumulation sustains MDSC function in murine models, suggesting metabolic disruption as a viable therapeutic approach (Kim et al., 2023).

#### 4.1.3 TAMs

Emerging scRNA-seq and spatial multi-omics technologies have revealed specialized TAM subpopulations that drive immune evasion, angiogenesis, and therapy resistance within the TME. Recent studies have revealed spatially restricted distributions of pro-tumoral (M1-like) and anti-tumoral (M2-like) TAM subsets, providing new opportunities for targeted intervention. Current interventions primarily focus on three mechanistic strategies. The first approach disrupts TAM recruitment by targeting chemokine axes, such as the CSF1-CSF1R pathway, where inhibitors like pexidartinib have demonstrated preclinical efficacy and are undergoing clinical assessment (Ries et al., 2014). CCL2-CCR2 axis blockade similarly reduces TAM infiltration while enhancing CD8^+^ T cell recruitment, improving antitumor immunity (Pittet et al., 2022). A second strategy reprograms TAM polarization, exemplified by CD40 agonists that convert M2-like TAMs to tumoricidal M1-like phenotypes—an approach showing synergistic effects with PD-1 blockade in clinical trials(NCT02376699) (Coveler et al., 2023). Additionally, inhibition of TREM2, a marker of lipid-metabolizing TAMs identified through omic analyses, has demonstrated the potential to enhance anti-tumor immunity (Kirschenbaum et al., 2024; Molgora et al., 2020). The third approach selectively depletes pro-tumoral subsets, including angiogenesis-associated SPP1+ TAMs and phagocytosis-impaired C1QC+ TAMs identified via multi-omics profiling (Cheng et al., 2021). Targeted therapies against these subsets may overcome resistance while preserving beneficial macrophage functions. Besides, the interaction between TAMs and blood vessels significantly promotes tumor growth, proliferation, and migration. Strategies such as co-targeting EGFR and VEGFR signaling pathways and inhibiting Notch signaling to block carcinogenic reprogramming may prove beneficial (Sharma et al., 2020).

### 4.2 Targeting non-cellular components: remodeling the ECM in the TME

Among ECM components, collagen forms fundamental structural networks that significantly influence drug distribution. Proteomic and imaging studies reveal that disseminated tumor cells remodel their microenvironment by forming type III collagen-enriched niches, sustaining dormancy via the DDR1-STAT1 signaling axis. Disrupting this pathway induces tumor reactivation, implying that ECM modulation may prolong dormancy and inhibit metastasis (Di Martino et al., 2022). Stromal profiling in pancreatic cancer datasets identifies LOXL2-rich niches as protective barriers in primary tumors, whereas LOXL2 depletion in liver metastases correlates with accelerated progression, warranting reassessment of anti-stromal therapies targeting collagen remodeling (Jiang et al., 2020).

Multi-omics integration has advanced the understanding of ECM-mediated resistance. Neuroendocrine prostate cancer adapts to therapy through tumor-specific ECM-driven epigenetic reprogramming, a vulnerability addressed by sequentially targeting epigenetic modifiers and DRD2 in synthetic organoids and xenografts (Mosquera et al., 2022). Furthermore, proteomics-defined metastasis-associated ECM signatures containing tenascin-C have enabled the development of tumor-agnostic nanobodies for targeted delivery of imaging and therapeutic agents, demonstrating the potential of ECM-guided precision targeting as evidenced by immuno-PET/CT validation in triple-negative breast and colorectal cancer metastases (Jailkhani et al., 2023). Single-cell multiomics in renal cell carcinoma implicates SERPINE2 as a metastatic regulator orchestrating EMT and tumor-TME crosstalk, suggesting its blockade could disrupt progression in advanced disease (Chen et al., 2023b).

Paradoxically, ECM softening promotes chemoresistance through DRP1/MIEF1/2-dependent mitochondrial fission and NRF2 activation, posing a therapeutic dilemma requiring nuanced targeting (Jain, 2014; Qin et al., 2020; Romani et al., 2022). Future investigations must delineate ECM-tumor interactions, refine ECM-directed strategies, and evaluate combinatorial approaches.

### 4.3 Targeting non-cellular components: remodeling the vascular reprogramming in the TME

Recent multi-omics studies have provided unprecedented insights into the mechanisms of vascular reprogramming in drug-resistant tumors and have identified novel therapeutic interventions for targeted vascular therapy. ScRNA-seq of brain metastasis vasculature uncovered CD276-enriched endothelial subtypes with immunoregulatory properties, and CD276 blockade improved survival in preclinical models, suggesting vascular immune checkpoint inhibition as a potential therapeutic strategy across multiple primary tumor types (Bejarano et al., 2024). Metabolomic profiling identified the sphingosine-1-phosphate receptor 1 (S1PR1)-STAT3-CerS3 signaling axis in endothelial cells as a driver of HCC angiogenesis through ceramide metabolism modulation, with S1PR1 inhibition showing synergistic effects when combined with Lenvatinib (Wang et al., 2022). Further omics analysis demonstrated that hypoxia-induced DGKG overexpression in HCC vascular endothelial cells, mediated by the USP16-ZEB2-TGF-β1 axis, facilitates immune evasion, while pathway inhibition enhances the efficacy of anti-PD-1/VEGFR2 therapy (Zhang et al., 2024).

These omics-derived insights are now informing novel clinical strategies. Dual-targeting agents, such as the bispecific anti-Ang2/VEGF-A antibody A2V, have shown promise in normalizing tumor vasculature while simultaneously reprogramming the TME (Kloepper et al., 2016). Multi-omics investigations of vascular-mediated drug resistance have yielded innovative approaches, including the FAP1V2 intracellular nanobody that concurrently targets PD-L1/PD-1 immune checkpoints and VEGFR2-driven metastasis, inducing durable tumor control through TCRβhi T-cell activation while preventing PD-1hi T-cell exhaustion (Zhang et al., 2025b). Hence, future research should focus on further elucidating the complex interplay between vascular reprogramming and the TME by using multi-omics approaches, developing more precise dual-targeting agents, and exploring the potential of rational combination therapies to enhance clinical outcomes.

### 4.4 Metabolic targeting in the TME

Multi-omics integration has revolutionized the design and optimization of metabolic therapies by systematically identifying actionable targets, predicting resistance mechanisms, and enabling precise patient stratification. Transcriptomic and metabolomic profiling of therapy-resistant tumors revealed consistent overexpression of glycolytic enzymes (GLUT1, HK2, PKM2), thereby nominating them as therapeutic targets (Liu et al., 2012; Feng et al., 2020). Single-cell multi-omics further validated HK2 as a biomarker for noninvasive bladder cancer detection, demonstrating 90% sensitivity and 98% specificity in urine-based liquid biopsies (Wang et al., 2020). This not only highlights the clinical utility of multi-omics in biomarker discovery but also underscores its potential for noninvasive cancer detection. Integrated genomics and metabolomics in lung cancer revealed BCKDK as an upstream regulator of MYC-dependent HK2 transcription, supporting dual BCKDK/HK2 inhibition to counter trametinib resistance (Wu et al., 2025).

Multi-omics approaches have clarified compensatory metabolic responses to targeted therapies, facilitating rational combination strategies. In castration-resistant prostate cancer, integrated transcriptomic and metabolomic data connected TP53 loss to ASNS-mediated asparagine dependence, informing the use of glutaminase inhibitors (e.g., CB-839) with L-asparaginase to induce synthetic lethality (Yoo et al., 2024). Proteomic flux analyses revealed that GLS inhibition triggers FAO/glycolysis upregulation (Choi and Park, 2018; Cheng et al., 2011), prompting clinical trials pairing GLS inhibitors (e.g., telaglenastat) with FAO blockers (e.g., etomoxir). Phosphoproteomic and lipidomic profiling in glioblastoma exposed an SREBP-1-ASCT2-glutamine resistance axis following lysosomal inhibition, guiding dual-targeting approaches (Zhong et al., 2024). Multi-omics has also been instrumental in connecting metabolic dysregulation to immunotherapy resistance. For instance, ARID1A-mutated ovarian cancers showed GLS1 dependency via metabolomics, motivating CB-839 + anti-PD-L1 trials (Wu et al., 2021b). Spatial metabolomics and transcriptomics identified BCAA metabolism (DLD/IL4I1-OXCT1 axis) and glutamine reprogramming as features of PD-1-high, therapy-resistant nasopharyngeal carcinoma, suggesting metabolic-immune co-targeting to address treatment resistance (Ji et al., 2025). Additionally, combining HK2 inhibitors with anti-PD-1 to reverse lactate-driven immunosuppression (Guo et al., 2022).

## 5 Discussion

Single-omics technologies have significantly advanced our understanding of tumor biology but remain insufficient for comprehensively characterizing the complex TME. Genomics fails to detect low-frequency variants, struggles with tumor purity, and cannot functionally annotate non-coding regions. Epigenomics encounters difficulties in establishing causal relationships due to low signal-to-noise ratios and sparse data coverage. Transcriptomics is constrained by the imperfect correlation between mRNA and protein levels, compounded by technical artifacts in single-cell RNA-seq. Proteomics currently captures only half of the human proteome, exhibits quantification variability, and misses transient molecular interactions. Metabolomics grapples with compound instability, methodological inconsistencies, and microbial contamination. These constraints underscore the imperative for multi-omics integration in TME research, as single-layer analyses cannot adequately capture cross-network communication or niche-specific signals obscured by bulk measurement limitations. Integrated multi-omics approaches overcome individual method weaknesses by uncovering higher-order biological relationships, distinguishing causal mechanisms from correlations, identifying compensatory pathways, and revealing non-cell-autonomous resistance patterns.

Multi-omics integration has revolutionized our comprehension of TME-mediated drug resistance, overturning conventional oncology paradigms. Where single-omics studies traditionally identified static biomarkers like PD-L1 expression for immune checkpoint blockade response, multi-omics uncovers context-dependent resistance networks—such as the requirement for CXCL13^+^ T cell niches to validate PD-L1 predictive value (Bassez et al., 2021)—and detects hybrid cellular states (e.g., EMT-metabolic intermediates in PDAC) invisible to single-layer analyses (Lin et al., 2020). However, key debates persist, including whether metabolic symbiosis mechanisms like the CAF-tumor lactate shuttle represent universal targets or context-dependent vulnerabilities (Ippolito et al., 2022). Tissue-level analyses obscure signals by averaging across heterogeneous cell populations, diminishing discovery potential and reproducibility. Advanced deconvolution algorithms using cell-type-specific reference signatures may address this limitation (Avila Cobos et al., 2020). The escalating volume of multi-omics data also necessitates optimized computational frameworks for multimodal integration. Although machine learning models show potential for improving clinical utility such as treatment response prediction (Sammut et al., 2022), obstacles remain in developing robust analytical pipelines and extracting biologically meaningful interpretations (Kang et al., 2022; Miao et al., 2021).

Three testable hypotheses may bridge these findings to clinical practice: First, spatial multi-omics can resolve discordance between tumor cell genotypes and TME phenotypes through predictable spatiotemporal phases, such as from fibrotic to immunosuppressive to metabolic states—detectable via integrated spatial transcriptomics, proteomics, and metabolomics, enabling novel target discovery (Peran et al., 2021). Second, cross-omics network analysis will reveal context-dependent resistance hubs—such as POSTN+ CAF/SPP1+ macrophage crosstalk via IL-6/STAT3—where combinatorial targeting (e.g., stromal-immune co-targeting) outperforms single biomarkers (Chen et al., 2023c). Third, Longitudinal integration of noninvasive dynamics (e.g., 2HG-MRS in gliomas, FDG-PET in breast cancer) with genomic clonal tracking can distinguish adaptive resistance(metabolic plasticity) from selection(subclone expansion), guiding mechanism-specific interventions (Suh et al., 2018; Zhou et al., 2018). Collectively, this framework links multi-omics layers to actionable clinical strategies while accounting for TME plasticity and evolutionary dynamics, thereby facilitating preoperative diagnosis, treatment monitoring, and recurrence surveillance.

Clinical translation of TME-targeting strategies faces substantial yet addressable barriers that require multi-omics-informed solutions. For instance, single-cell RNA-seq reveals niche-specific VEGF expression patterns that demand spatially precise delivery systems, with emerging solutions including MMP2-responsive nanoparticles and spatial multi-omics-guided combinations (Huang et al., 2013b). Adaptive resistance mechanisms, such as therapy-induced kynurenine accumulation, require real-time metabolite tracking coupled with preemptive pathway inhibition. Historical failures emphasize the critical need for proteomic validation in target prioritization. Emerging solutions include modular clinical trials testing stromal disruptors combined with immune modulators and “digital twin” approaches pairing patient-derived organoids with multi-omics simulations. To overcome these hurdles, innovative trial designs are emerging, including modular platform trials testing stromal disruptors with ICIs and “digital twin” approaches that integrate patient-derived organoids with longitudinal multi-omics profiling and AI-based treatment response prediction. These solutions directly address the translational challenges while leveraging multi-omics integration to develop clinically actionable strategies.

## 6 Conclusion

Therapeutic resistance represents a complex and evolving challenge in cancer treatment, with the TME serving as a central and dynamic contributor. As our understanding of TME-mediated resistance continues to deepen, the integration of multi-omics technologies offers a powerful lens through which to uncover hidden regulatory networks and guide precision intervention. Shortly, leveraging multi-omics insights to develop context-specific, combinatorial strategies that modulate the TME in real-time will be critical. Continued efforts toward translating these findings into clinically actionable approaches hold promise for overcoming resistance and improving durable patient outcomes.

## Glossary

ABC: ATP-binding cassette
AR: androgen receptor
ATAC-seq: transposase-accessible chromatin using sequencing
CAF: Cancer-associated fibroblast
ChIP-seq: chromatin immunoprecipitation sequencing
CSCs: cancer stem sells
CTL: cytotoxic T lymphocyte
CTLA-4: Cytotoxic T-lymphocyte–associated protein 4
CPT1B: carnitine palmitoyltransferase 1B
D-2-HG: D-2-hydroxyglutarate
ECM: Extracellular matrix
EGFR: Epidermal growth factor receptor
EMT: epithelial-mesenchymal transition
EVs: extracellular vesicles
FAO: Fatty acid oxidation
GM-CSF: Granulocyte-macrophage colony-stimulating factor
HCC: hepatocellular carcinoma
HK2: hexokinase-2
ICIs: Immune checkpoint inhibitors
LC-MS/MS: liquid chromatography-tandem mass spectrometry
MIF: migration inhibitory factor
MALDI-MSI: matrix-assisted laser desorption/ionization mass spectrometry imaging
MDSC: Myeloid-derived suppressor cell
NMR: Nuclear magnetic resonance
NK cell: Natural killer cell
NMR: nuclear magnetic resonance
NSCLC: Non-small cell lung cancer
PD-1: Programmed cell death protein 1
PD-L1: Programmed death-ligand 1
PDGF: Platelet-derived growth factor
PDAC: pancreatic ductal adenocarcinoma
PTMs: post-translational modifications
P-gp: P-glycoprotein
PFK1: phosphofructokinase-1
PK: pyruvate kinase
scRNA-seq: Single-cell RNA sequencing
TAM: Tumor-associated macrophage
TCA: Tricarboxylic acid cycle
TGF-β: Transforming growth factor-beta
TLS: tertiary lymphoid structures
TMB: High tumor mutation burden
TNBC: triple-negative breast cancer
TME: tumor microenvironment
Treg: Regulatory T cell
VEGF: Vascular endothelial growth factor
WES: whole-exome sequencing
WGS: whole-genome sequencing
ZEB2: Zinc Finger E-Box-Binding Homeobox 2
2-DG: 2-deoxy-D-glucose
